# A Pilot Study of Functional Outcomes in Adult Patients Treated With Valgus Intertrochanteric Osteotomy as a Primary Modality for Intracapsular Femoral Neck Fracture

**DOI:** 10.7759/cureus.60205

**Published:** 2024-05-13

**Authors:** Arjun S Chakrapani, Rahul Ragate, Arfaz Shaik, Varun Thusoo, Mukul Kumar Singh, Abhishek Nair, Amnish Kumar, Ritesh Jadav

**Affiliations:** 1 Orthopaedics, Apollo Speciality Hospitals, Chennai, IND; 2 Orthopaedics, Sir H. N. Reliance Foundation Hospital and Research Centre, Mumbai, IND; 3 Orthopaedics, Wrightington, Wigan and Leigh NHS Foundation Trust, England, GBR; 4 Orthopaedic Surgery, Adesh Medical College and Hospital, Ambala, IND; 5 Orthopaedics, Rajiv Gandhi Institute of Medical Sciences, Adilabad, IND

**Keywords:** implants, avascular necrosis, radiography, osteotomy, femoral neck fracture

## Abstract

Background

Intracapsular femoral neck fractures account for a majority of hip fractures. This study aimed to investigate the efficacy of valgus osteotomy as a primary treatment for intracapsular femoral neck fractures in adult patients aged 15-60 years, assessing its impact on functional outcomes and fracture union.

Methodology

A retrospective clinical analysis was conducted at the Department of Orthopedics and Traumatology, Osmania Government General Hospital, Hyderabad, India, focusing on patients treated with primary intertrochanteric valgus osteotomy for intracapsular femoral neck fractures. The study reviewed medical charts and radiographs of six patients aged between 15 and 60 years, diagnosed with recent isolated intracapsular femoral neck fractures, presenting between May 2019 and October 2021. The intervention involved Pauwels' intertrochanteric valgus osteotomy with various fixation methods. Main outcome measures included radiographic union, functional ability assessed by the Harris Hip Score, and evaluation for complications.

Results

All six patients achieved radiographic union at fracture and osteotomy sites, totaling a 100% success rate. The average follow-up duration was 14.8 months (12-20 months), with an average time of 5.1 months (2.5-6 months) from surgery to radiographic union. One patient experienced union with retroversion, while another developed avascular necrosis (AVN) by the study's conclusion. No instances of hardware failure or non-union were observed. The average Harris Hip Score obtained during the most recent clinical follow-up was 84, ranging from 69 to 94. All six patients regained independent walking ability without any support by the end of the follow-up period.

Conclusion

The combination of primary Pauwels' intertrochanteric valgus osteotomy with fixed-angle plating proves to be a highly effective method for addressing recent intracapsular femoral neck fractures, resulting in a 100% success rate in achieving union among the patient cohort.

## Introduction

The femoral neck fracture has earned the moniker "the unsolved fracture" due to the absence of a universally successful treatment despite advancements in orthopedics [1,2]. Managing this fracture, particularly in younger patients, presents a daunting and challenging endeavor [3]. Intracapsular femoral neck fractures constitute about half of all hip fractures [4], with non-union rates reaching up to 30%, varying according to the precise location of the fracture [5].

Femoral neck fracture predominantly affects the elderly, particularly those with osteoporosis, given their increased susceptibility [6]. However, with advancements in quality of life and subsequent rise in life expectancy, the incidence of this fracture has become even more prevalent in recent times. Additionally, it is frequently observed in younger patients following road traffic accidents (RTA), often accompanying polytrauma scenarios.

Because of the femoral neck's unique blood supply, many of these fractures show instability [7], which poses severe dangers since it can cause circulatory disturbances that can result in avascular necrosis (AVN) and non-union [8]. Consequently, every femoral neck fracture must be treated as an emergency [7], with precise and anatomical reduction and stable fixation in those without predisposing physiological impediments to osteosynthesis, with one of the several implants that are available. Undisplaced stable fractures are often associated with better prognoses, while displaced unstable fractures are linked to worse outcomes [9].

The angle of inclination of the fracture is a key contributing factor to non-union [4,9]. Horizontal fractures with angles less than 30 degrees often unite successfully, while those with angles more than 30 degrees are predisposed to non-union even with skilled care. This condition happens due to shearing forces acting on fractures with angles greater than 30 degrees, which produce fragment displacement and eventual non-union [9].

The enduring relevance of Pauwels' principle, first articulated in 1927, persists in contemporary orthopedics and has been successful in its application [7]. According to this principle, femoral neck fractures can achieve union when the fracture inclination angle is altered to convert shearing forces into compression forces [10-12]. This converts unstable fractures into stable ones and is achieved primarily by performing a valgus intertrochanteric osteotomy, though other osteotomies have also been described [13]. Though there is extensive literature available on the use of Pauwels' osteotomy and other such osteotomies to treat non-union intracapsular femoral neck fractures [10,11], the literature regarding the use of this osteotomy to manage fresh intracapsular femoral neck fractures is sparse [13-17].

The ultimate goal of treating a femoral neck fracture is to attain healing of the patient's own femoral neck and retain a painless biological hip joint, and every effort should be directed toward achieving this objective. The present study aims to retrospectively examine the outcomes of six patients who underwent Pauwels' intertrochanteric valgus osteotomy as the primary approach for managing recent intracapsular femoral neck fractures.

## Materials and methods

Study design and setting

This retrospective clinical analysis was conducted at the Department of Orthopedics and Traumatology, Osmania Government General Hospital, Hyderabad, India, focusing on evaluating outcomes of patients treated with primary intertrochanteric valgus osteotomy for intracapsular femoral neck fractures through the review of patient's medical charts and radiographs. It was conducted as a pilot project to conduct a large-scale study in the future. Consent was obtained or waived by all participants in this study. The Ethics Committee of Osmania Medical College issued approval ECR/300/Inst/AP/2013/RR-19.

Participants and measurements of variables 

Individuals who had established non-union, concomitant ipsilateral intertrochanteric fractures, or radiological or clinical signs of AVN of the femoral head were excluded. Two male and four female patients were selected for the pilot, with an age range of 25-50 years.

**Figure 1:**
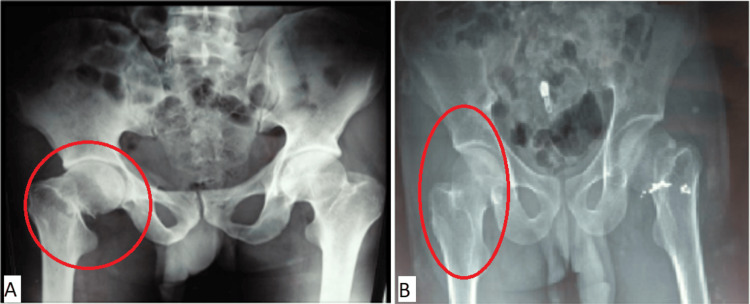
Radiographic examination of two of the patients with intracapsular femoral neck fractures (A) A 25-year-old male having a fracture due to RTA. (B) A 45-year-old female having a fracture due to a self-fall RTA: road traffic accident

Between May 2019 and October 2021, six patients (10% of the original sample size of 60) between the ages of 25 and 50 years with a recent (three-day- to one-month-old) intracapsular femoral neck fracture (Figure 1) presented to the Department of Orthopedics and Traumatology at Osmania Government General Hospital, Hyderabad, and subsequently underwent an open reduction and internal fixation (ORIF) with Pauwels' intertrochanteric valgus osteotomy using a fixed-angle implant such as a 120-degree double-angled blade plate with screw, a 120-degree double-angled dynamic condylar screw (DCS), or a 120-degree double-angled dynamic compression plate (DCP). This technique was previously described by Pauwels [7] and further documented by Marti et al. [11].

Surgical Phase 

**Figure 2:**
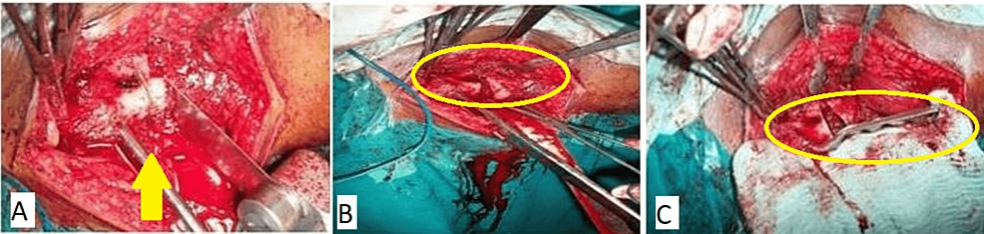
Intra-operative images (A) Seating chisel, osteotomy, and wedge. (B) After wedge removal. (C) Reduction with osteotomy and fixation with 120-degree double-angled plate

Pre-operative planning was crucial, involving considerations such as blade placement, osteotomy level, and the amount of bone wedge excision required [7,15]. An anterior-posterior hip X-ray was taken during this procedure while the leg was under traction, and internal rotation ranged from 10 to 20 degrees. On tracing paper put over the X-ray, the acetabulum, head, neck, proximal third of the femur, and angle of fracture inclination were traced. The surgical objective was to achieve a 30-degree inclination of the fracture line to convert shearing into compressive forces across the fracture site. Calculations for the wedge excision were based on subtracting 30 degrees from the initial fracture inclination. A horizontal line for osteotomy was marked at the upper border of the lesser trochanter perpendicular to the femur axis. A lateral-based wedge with the calculated angle was delineated below this line, with wedges of up to 30 degrees taken below the lesser trochanter. In one case requiring a 45-degree wedge, the initial 30-degree wedge was taken below the lesser trochanter and the remaining amount above it. A cortical bridge of around 15 mm was kept in place between the osteotomy site and the seating chisel's insertion point. The distance between the blade and the plate cutback identified the blade's entrance location in the lateral femoral cortex above the osteotomy. With the use of known angular dimensions during surgery, the required wedge angle in the coronal plane was used to calculate the angle of blade entrance with respect to the shaft. The angle of blade entrance with respect to the femoral shaft for a double-angled blade plate with a 120-degree angle was computed as 180 degrees plus the appropriate wedge angle minus 120 degrees. The calculated angle of adjustment would be 60 degrees plus the target angle. Comparable concepts were employed while employing DCP and DCS with a 120-degree double angle. Figure 2 shows the intra-operative images showing the procedure in three different stages (panels).

Follow-Up Phase

All patients underwent hip mobilization following surgery with non-weight-bearing hip and knee exercises for up to six weeks. Formal therapy commenced once radiographic signs of union were observed. During routine post-operative visits at the second, fourth, sixth, and eighth weeks, as well as at the six-month mark, patients were evaluated by the primary author through interviews, physical examinations, and surveys. The Harris Hip Score was calculated at each visit based on the gathered information. Additionally, subjective assessments of pain and satisfaction were obtained using a Visual Analog Scale administered at each routine appointment.

Data analysis

As a pilot project, extensive data sets were not generated enough to apply robust statistical tests. Data collection was done using the Kobo Toolkit, wherein the pre-surgical, post-surgical, and follow-up details were stored for validating future projects. The Harris Hip Score was maintained during the follow-up visits of the patients. 

## Results

After initial care at the Osmania Government General Hospital, the same treating specialist operated on each patient. For two patients, it was a traffic accident; for the remaining four, it was a self-fall. Four patients had 120-degree double-angled blade plates and screws, one patient had 120-degree double-angled DCS, and one patient had 120-degree double-angled DCP plates. This was the strategy used to handle these patients. Four individuals had Pauwels' type III fractures, and two had Pauwels' type II fractures [14]. The same has been depicted in Table 1. 

**Table 1:** Study results of the pilot M: male; F: female; RTA: road traffic accident; DCP: dynamic compression plate; DCS: dynamic condylar screw; AVN: avascular necrosis

Sr. no.	Age (in years)	Sex	Injury	Pauwels	Fixation mode	Fracture	AVN	Follow-up	Harris Hip Score	Result
1	50	F	Self-fall	II	120-degree double-angled plate + screw	6 months	Yes	12 months	69	Poor
2	32	M	RTA	III	120-degree double-angled DCS	9 months	No	20 months	94	Excellent
3	45	F	Self-fall	II	120-degree double-angled blade plate	6 months	No	12 months	86	Good
4	25	M	RTA	III	120-degree double-angled DCP	6 months	No	18 months	93	Excellent
5	28	F	Self-fall	III	120-degree double-angled blade plate	5 months	No	12 months	90	Excellent
6	50	F	Self-fall	III	120-degree double-angled blade plate	5 months	No	15 months	74	Fair

**Figure 3:**
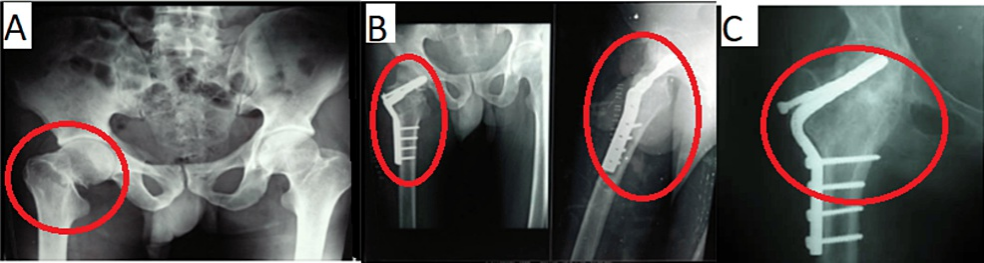
Pre- and post-operative radiographs with radiographic union (A) Pre-operative. (B) Six-month follow-up. (C) Twenty-month follow-up

With the present surgical method, 100% of the patients had radiographic union at fracture and osteotomy sites. This was the case for six out of six patients. The follow-up period was 14.8 months on average (range: 12-20 months). From surgery to the radiographic union, it took an average of 5.1 months (range: 2.5-6 months). The average operative time was 151 minutes. One patient had a union with retroversion. One patient developed AVN by the end of the study period, and there were no cases of hardware failure or non-union. There were no cases of post-operative infection. Five patients had a normal gait, and one had a moderate limp. Five patients had more than 75% range of movements compared to the other hip, whereas one patient had a 60% range of motion (ROM). Five of the six patients could carry out all daily activities, including walking, cycling, squatting, and sitting cross-legged, while one found it challenging. One patient experienced 1 cm shortening, whereas five individuals showed no difference in limb length. Figure 3 shows the radiographic images of a patient pre-operation, six-month follow-up, and 20-month follow-up stages. 

Stiffness was noted in one patient. The average Harris Hip Score measurements obtained from the most recent clinical follow-up were 84 (range 69-94), which equates to good (>90 excellent, 80-90 good, 70-80 moderate, <70 poor). All six patients could walk without support by the end of the follow-up period. The average pain on the Visual Analog Scale at six-month follow-up was 1 compared to 8 pre-operatively. Five out of six patients reported they were satisfied with the result of the operation.

## Discussion

Instead of a prosthesis, the patient's repaired femoral neck would be the ideal outcome following a femoral neck fracture [18]. This is because the former maintains stability and a complete ROM in various joint postures, including those involved in cross-legged sitting, and is particularly crucial for Indian patients. Pauwels' osteotomy seeks to attain this outcome through the manipulation of the biomechanical environment of the fracture [19-23]. Our series consists of six patients with a fresh intracapsular femoral neck fracture (defined as any fracture under one-month-old in our study with no clinical or radiological signs of AVN) in the age group range of 16-60 years. We achieved union in six out of six cases, or 100%. One of the patients developed AVN after union. Our series is small, which was the primary deficiency identified in our study.

In a study by De Palma in 1949 [16], 100% union was achieved in a series of 22 patients aged between 62 and 83 years with fresh adduction-type intracapsular femoral neck fractures treated by valgus wedge osteotomies fixed with Smith-Petersen nails [16]. Nonetheless, our analysis identified two cases of femoral head AVN. In 1979, Mishra [24] published a report on 51 patients with intertrochanteric displacement osteotomy for newly displaced femoral neck fractures of Garden grade III or IV. Of them, 31 fractures (73.8%) united, and 42 patients had complete follow-up. Outcomes were good in 66.6% of patients and bad in 33.4%; non-union, persistent osteomyelitis, and stiffness in the hip or knee were the reasons for the latter [24-26]. In 1984, Rinaldi et al. [22] published a report on 25 consecutive occurrences of femoral neck sub-capital fractures, most of which were treated with valgus osteotomy and osteosynthesis. Findings at three to five years revealed that all individuals had a bone union, with just two cases (8%) of ischemia necrosis noted.

Magu et al. in 2005 reported outcomes on 50 adult patients with osteoporosis who underwent primary valgus intertrochanteric osteotomy for displaced femoral neck fractures. They achieved a 94% fracture union rate with an average union time of 12.2 weeks, with 100% union observed at the osteotomy site. The average neck shaft angle reached was 141 degrees, and 28% of patients had post-operative femoral head retroversion. There was an AVN rate of 8%. Final results showed that 76% of patients had outstanding to exceptional results (average Harris Hip Score 92), 18% had reasonable results (average Harris Hip Score 73), and 6% had poor results (average Harris Hip Score 30). Around 2% of patients had a deep infection, 4% had a superficial infection, 4% had an implant penetration into the joint, 6% had a limb length disparity, and 68% had an external rotation [21].

Notably, none of the patients in their series experienced implant cut-through, possibly attributed to adequate fracture reduction and avoidance of premature weight bearing. It's important to note that because of the high bending stresses in this area and the often low bone quality associated with fractures, implants utilized for internal fixation of the proximal femur are prone to failure.

In our study, the outcome was affected by AVN, which was observed in one case. If more AVN diagnostic techniques are used, or if all patients are monitored for a more extended period, this rate can rise. Our AVN rate of 16%, however, is not higher than the numbers published in the literature after femoral neck fractures, which mention AVN rates ranging from 12% to 55% that have been verified by radiographic and scintigraphical means [2,25]. According to some writers, longer healing times may increase the incidence of AVN in femoral neck fractures or non-unions. However, Marti et al. discovered no association between the functional results measured by the Harris Hip Score and the time between the initial fracture and the osteotomy [11].

In a study by Brümmer, mild AVN was observed in five out of 17 cases [26]. One of the patients in our investigation showed AVN changes throughout time, and at the end of the study period, Ficat and Arlet stage 2 AVN was diagnosed. The bulk of vascular insults happen at the time of the first injury. However, AVN is impacted by several factors, including the method of damage, the amount of time that passes between the injury and surgery, trauma during surgery, and the initial injury to retinacular arteries. It has been demonstrated that valgusisation up to 30 degrees has no negative impact on the retinal arteries.

Patients automatically assumed postures that alleviated intra-articular tamponade, such as flexion, abduction, and external rotation, hence lowering the incidence of AVN, as noted by Soto Hall et al. AVN was infrequent when therapy was disregarded. In situations of extensive AVN, implant cut-through, and failed valgus osteotomy, total hip replacement (THR) is recommended. However, THR after proximal femur osteotomy requires careful planning ahead of time, careful implant selection, and cautious surgical technique. For this reason, fears regarding valgus osteotomy's potential to reduce the outcomes of a THR thereafter could be a reason it is not generally considered a therapeutic option for femoral neck fractures [27,28]. In our series of six patients, none experienced femoral head collapse or secondary hip joint osteoarthritis. Although one patient did develop AVN of the femoral head (Ficat and Arlet stage 2), they would likely undergo THR before head collapse occurs.

A stable medial buttress is absent from many displaced fractures, characterized by posterior and medial comminution, making them unstable. In these situations, implant complications can occur, such as peri-implant fracture, implant failure and breakage, loosening from the femoral shaft in the case of a side plate device, or cut-out from the superior femoral neck when the fracture settles into a varus position. Failure can be caused by technical problems such as incorrect fracture reduction and poorly positioned blades. Out of 28 valgus osteotomies, Alami-Harandi and Norouzi's research found two implant failures [29]. Similarly, Brümmer noted three instances where implant failure at the shaft or perforated blades necessitated revision osteosynthesis and implant exchanges [26].

Limitations

This pilot study on valgus osteotomy for intracapsular femoral neck fractures has several limitations, including a small sample size of only six patients, which limits the generalizability of the results. Its retrospective design may introduce selection and recall biases. The study's short follow-up period of less than two years may not adequately capture long-term outcomes or complications such as AVN, which could develop later. Finally, the lack of a control group prevents comparison with other treatment modalities, which can be delved into future studies. As a pilot study, the aim was to generate a fresh interest in this treatment modality and pave the way for a larger study in the same institute in the future. 

## Conclusions

For patients under 60 years of age with fresh intracapsular femoral neck fractures, Pauwels' osteotomy yields favorable outcomes. The current technique has demonstrated a safe and reliable means of achieving union in 100% of patients while circumventing the potential pitfalls associated with hip arthroplasty. Further validation of these findings through more extensive series and more extended follow-up periods would be beneficial to solidify the technique's efficacy and promote its wider adoption as a primary treatment modality for these fractures in patients under 60.
